# Gα_i3_ signaling is associated with sexual dimorphic expression of the clock-controlled output gene *Dbp* in murine liver

**DOI:** 10.18632/oncotarget.25727

**Published:** 2018-07-13

**Authors:** Madhurendra Singh, Laura Bergmann, Alexander Lang, Katja Pexa, Fabian Kuck, Dennis Stibane, Linda Janke, Hakima Ezzahoini, Antje Lindecke, Constanze Wiek, Helmut Hanenberg, Karl Köhrer, Charlotte von Gall, Hans Reinke, Roland P. Piekorz

**Affiliations:** ^1^ Institut für Biochemie und Molekularbiologie II, Medizinische Fakultät der Heinrich-Heine-Universität, Düsseldorf, Germany; ^2^ Biologisch-Medizinisches Forschungszentrum (BMFZ), Medizinische Fakultät der Heinrich-Heine-Universität, Düsseldorf, Germany; ^3^ Hals-Nasen-Ohren-Klinik, Medizinische Fakultät der Heinrich-Heine-Universität, Düsseldorf, Germany; ^4^ Institut für Anatomie II, Medizinische Fakultät der Heinrich-Heine-Universität, Düsseldorf, Germany; ^5^ Institut für Klinische Chemie und Laboratoriumsmedizin, Medizinische Fakultät der Heinrich-Heine-Universität, Düsseldorf, Germany; ^6^ IUF – Leibniz Institut für Umweltmedizinische Forschung, Düsseldorf, Germany; ^7^ Klinik für Kinderheilkunde III, Universitätsklinikum Essen, Universität Duisburg-Essen, Essen, Germany; ^8^ Current address: Department of Microbiology, Tumor and Cell Biology, Karolinska Institute, Stockholm, Sweden

**Keywords:** circadian regulation, galphai3/GNAI3, CREB, cytochrome P450, albumin D-box binding protein, Pathology

## Abstract

The albumin D-box binding protein (DBP) is a member of the PAR bZip (proline and acidic amino acid-rich basic leucine zipper) transcription factor family and functions as important regulator of circadian core and output gene expression. Gene expression of DBP itself is under the control of E-box-dependent binding by the Bmal1-Clock heterodimer and CRE-dependent binding by the cAMP responsive element binding protein (CREB). However, the signaling mechanism mediating CREB-dependent regulation of DBP expression in the peripheral clock remains elusive. In this study, we examined the role of the GPCR (G-protein-coupled receptor)/Gα_i3_ (Galphai3) controlled cAMP-CREB signaling pathway in the regulation of hepatic expression of core clock and clock-regulated genes, including *Dbp*. Analysis of circadian gene expression revealed that rhythmicity of hepatic transcript levels of the majority of core clock (including Per1) and clock-regulated genes were not affected by Gα_i3_ deficiency. Consistently, the period length of primary Gα_i3_ deficient tail fibroblasts expressing a Bmal1-Luciferase reporter was not affected. Interestingly, however, Gα_i3_ deficient female but not male mice showed a tendentiously increased activation of CREB (nuclear pSer133-CREB) accompanied by an advanced peak in *Dbp* gene expression and elevated mRNA levels of the cytochrome P_450_ family member Cyp3a11, a target gene of DBP. Accordingly, selective inhibition of CREB led to a strongly decreased expression of DBP and CYP3A4 (human Cyp3a11 homologue) in HepG2 liver cells. In summary, our data suggest that the Gα_i3_-pCREB signalling pathway functions as a regulator of sexual-dimorphic expression of DBP and its xenobiotic target enzymes Cyp3a11/CYP3A4.

## INTRODUCTION

The circadian clock system in mammals is hierarchically organized comprising a central master clock and peripheral clocks [1, 2]. The central clock localizes to the suprachiasmatic nuclei (SCN) of the anterior hypothalamus and is mainly synchronized by environmental light [3, 4]. In contrast, synchronization of peripheral clocks, which are present in nearly all tissues and organs, occurs by hormonal and neuronal cues and/or food intake [5–7]. In central and peripheral clocks, the molecular clockwork is composed of intertwined positive and negative feedback loops at the transcriptional and (post)translational level [4, 8–10]. In particular, core molecular clockwork components encoded by so-called clock genes regulate positively (Clock, Bmal1) or negatively (Cry1/Cry2 and Per1/Per2) rhythmic expression of their target genes (i.e., clock output genes) through transcriptional control *via* so-called E-box elements [11]. Important clock output genes include members of the family of PAR-domain basic leucine zipper (PAR bZip) transcription factors, i.e. hepatic leukemia factor (HLF), thyrotrophic embryonic factor (TEF), and albumin D-box binding protein (DBP) [12]. The latter activates gene expression of Per1/2 *via* DBP binding sites (so-called D-box elements) therefore modulating the levels of core clock gene products [13, 14]. Moreover, PAR bZip proteins control the circadian expression of hepatic enzymes and regulators involved in endobiotic and xenobiotic biotransformation and drug metabolism [15], including isoenzymes of the cytochrome P450 family of monooxygenases.

Several findings indicate an important role of the G_s_/G_i_-protein-controlled cAMP-CREB signaling pathway in the regulation of clock gene expression. Treatment of Rat-1 fibroblasts with forskolin, which activates adenylyl cyclase and therefore increases intracellular cAMP concentrations, resulted in elevated pSer133-CREB levels concomitant with an enhanced circadian accumulation of DBP, Per1, and Per2 [16]. Moreover, the cAMP-CREB signaling pathway positively regulates Per1 expression in human hepatoma cells [17] as well as the light-induced Per1 expression in the SCN [18, 19]. In the SCN, CREB is rapidly phosphorylated at Ser133 after light at night [20–22], but circadian oscillation of intracellular cAMP is also important for intercellular coupling of SCN neurons [23]. In liver, intracellular cAMP concentrations and CREB activation are modulated by Cry1 [24].

Signaling by GPCRs *via* heterotrimeric G_s_ (i.e., Gα_s_βγ) and G_i_ (i.e., Gα_i_βγ) proteins activates or inhibits, respectively, cAMP production by adenylyl cyclases, thereby regulating downstream cAMP-PKA (protein kinase A)-CREB signaling [25]. The Gα_i_ subunits comprise three highly homologous isoforms, Gα_i1_, Gα_i2_, and Gα_i3_ [26, 27], which display a high amino acid sequence identity together with overlapping as well as cell- and tissue-type specific expression profiles. Overall, Gα_i2_ represents the predominantly and ubiquitously expressed isoform, whereas Gα_i3_ is found in all peripheral tissues with only low detectable expression in neuronal cells [28–30]. Of note, both Gα_i2_ and Gα_i3_ are expressed in the liver where Gα_i3_ exhibits an isoform-specific crucial function in the anti-autophagic action of insulin in hepatocytes [29]. However, the function of pertussis toxin-sensitive G_i_ proteins in circadian signaling and gene expression is largely unclear.

Here, we analyzed the role of Gα_i3_ in rhythmic pSer133-CREB activation and clock gene expression in the liver in mice. The phase of clock genes Per1, Per2, Bmal1, Rev-Erba, Cry1, and Cry2 as well as clock output genes Hlf and Tef was not different between *Gα_i3_^−/−^* and wild-type littermates of both genders. In contrast, in *Gα_i3_^−/−^* females the phase of the clock output gene DBP was advanced. This was associated with a slight increase in pCREB and DBP protein levels in females. Moreover, the expression level of the DBP target gene and cytochrome P450 family member Cyp3a11 were significantly higher in females at mid-day and mid-night, and in males at early day. Thus, our data indicate that the Gα_i3_-pCREB signaling pathway functions as a novel direct (*via* DNA binding) or indirect regulator of sexual-dimorphic hepatic expression of DBP and its xenobiotic targets.

## RESULTS

### Rhythmic clock gene expression is not affected by Gα_i3_ deficiency

**Figure 1:**
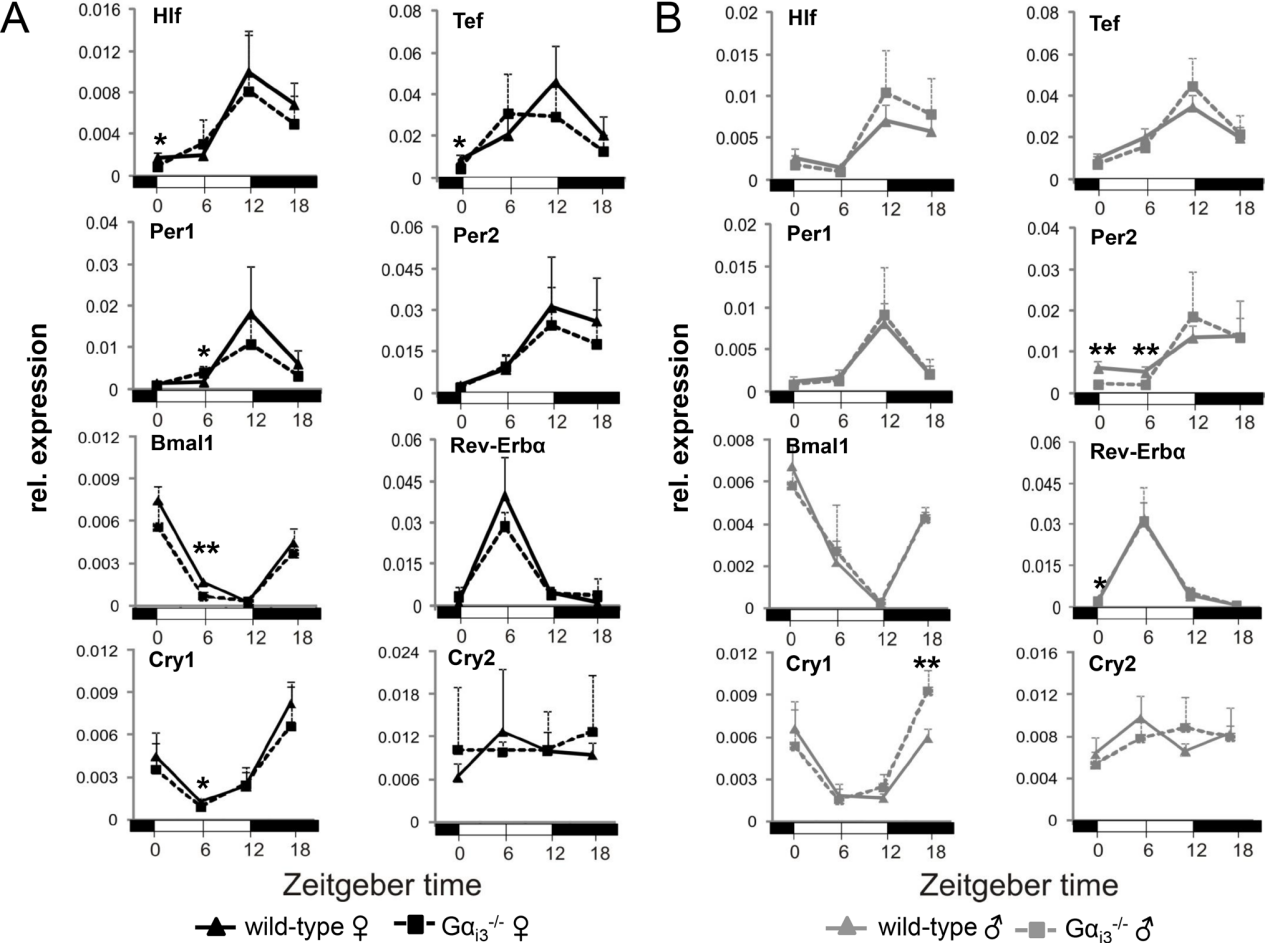
Rhythmic expression of core clock genes and clock-regulated genes in the liver of *Gα_i3_*^-/-^ mice Quantitative real-time PCR analysis of rhythmic expression of core clock genes and clock output genes in the liver of female (**A**) and male (**B**) Gα_i3_ deficient mice *vs*. wild-type control animals. Mice were sacrificed and analyzed every six hours at the indicated time points (ZT 0 to ZT 18). Transcript levels of the indicated genes were normalized to the endogenous control Gapdh. Shown are 2^-ΔCt^ values. Results are expressed as mean ± s.d. of six animals analyzed per genotype and time point (^*^p < 0.05; ^**^p < 0.01 as compared to corresponding wild-type control animals).

The cAMP-CREB signaling pathway, that is under the control of heterotrimeric G_s_ and G_i_ proteins, regulates the expression of core clock and clock-regulated genes, including Per1 and DBP [16, 17]. To characterize the role of the G*α*_i_ isoform Gα_i3_ in this pathway, Gα_i3_ deficient mice [29] and wild-type control animals were kept in a 12 h light/12 h dark cycle setting and liver tissue was collected every 6 h for up to 24 h followed by gene expression analysis *via* quantitative real-time polymerase chain reaction (qRT-PCR). As depicted in Figure 1, the phases of rhythms in transcript levels of the core molecular clockwork components (*Per1*, *Per2*, *Bmal1*, *Rev-erba*, *Cry1*, and *Cry2*) and clock-regulated genes (*Hlf* and *Tef*) and their expression levels were comparable between *Gα_i3_*^−/−^ mice and wild-type controls of both genders. In male *Gα_i3_*^−/−^ mice, the peak in *Cry1* expression at ZT18 was significantly higher as compared to wild-type controls (Figure 1B). To address these molecular findings at a functional level, we next isolated primary tail fibroblasts from wild-type and *Gα_i3_*^−/−^ female mice followed by lentiviral transduction of a Bmal1 promoter-driven luciferase reporter construct and subsequent real-time bioluminescence monitoring. As indicated in Supplementary Figure 1A, and consistent with the expression profiling results in Figure 1, recording of luciferase activity revealed a circadian period length that was nearly identical between Gα_i3_ deficient fibroblasts and wild-type cells (25.1 *vs.* 25.0 h, respectively; Supplementary Figure 1B).

### Gα_i3_ deficient female mice exhibit a phase-shifted rhythm of hepatic DBP expression

**Figure 2:**
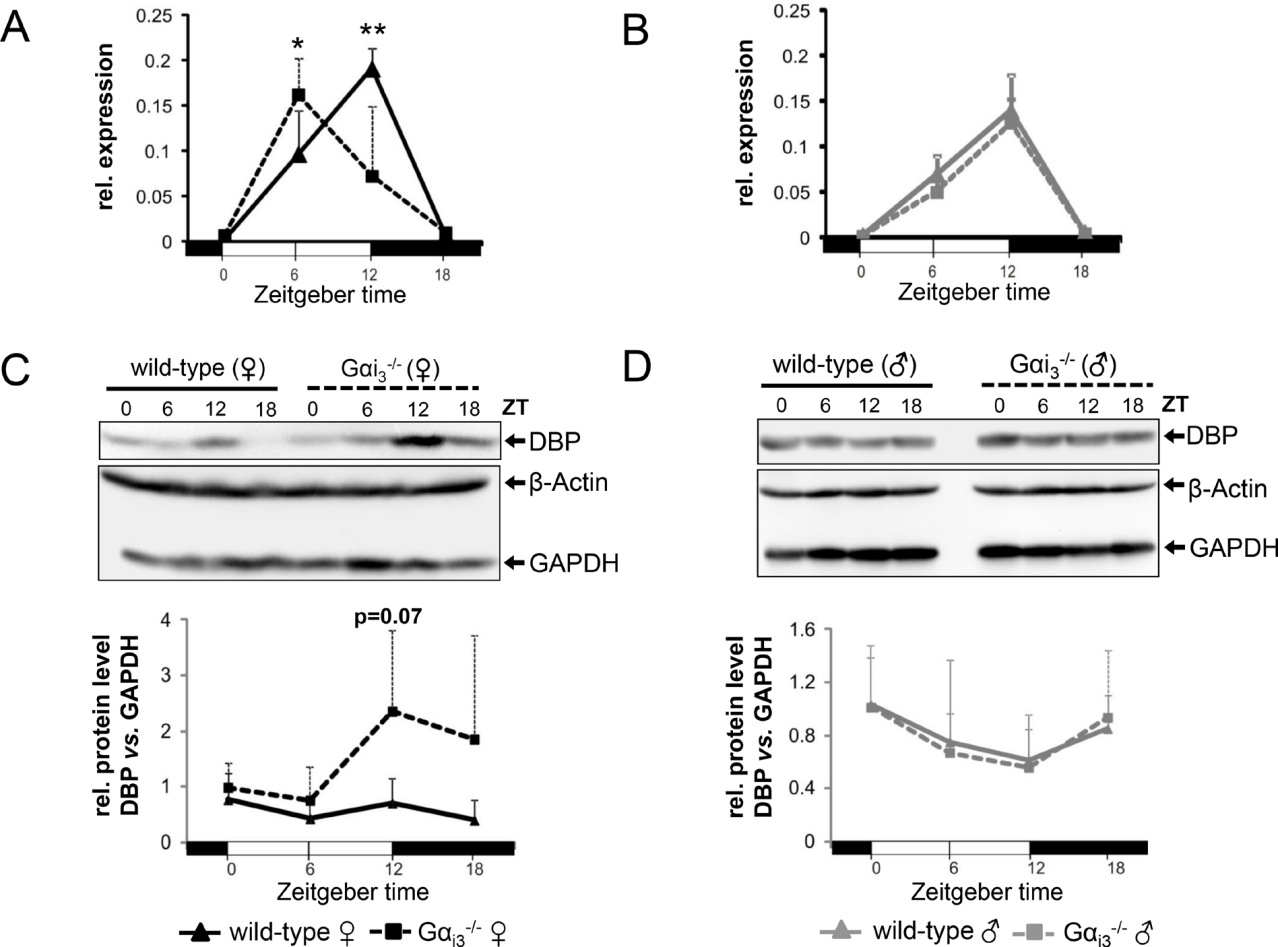
Rhythmic expression of DBP in the liver of female Gα_i3_^-/-^ mice is phase advanced (**A**, **B**) Quantitative real-time PCR analysis of rhythmic expression of DBP mRNA in the liver of female (A) and male (B) Gα_i3_ deficient mice as compared to wild-type control animals. Dbp transcript levels were normalized to the endogenous control Gapdh. Shown are 2^-ΔCt^ values. (**C**, **D**) Representative immunoblots of rhythmic expression of the DBP protein in the liver of female (C) and male (D) Gα_i3_-deficient mice and wild-type controls. GAPDH and β-Actin were employed as loading controls. Relative protein levels (DBP/GAPDH) were determined by densitometric analysis using ImageJ software (lower panels in C and D). Mice were sacrificed and analyzed every six hours at the indicated time points (ZT 0 to ZT 18). Results are expressed as mean ± s.d. of six animals (mRNA) or four animals (immunoblot) analyzed per genotype and time point (^*^p < 0.05; ^**^p < 0.01 as compared to corresponding wild-type control animals).

Interestingly, in contrast to the clock output genes and PAR bZip members Tef and Hlf, whose transcript levels were apparently not influenced by Gα_i3_ deficiency (Figure 1A), rhythmic expression of the PAR bZip member DBP was phase advanced by six hours in *Gα_i3_^−/−^* females as compared to wild-type females (Figure 2A and Supplementary Figure 2A). Here, comparable results were obtained using Gapdh, β-Actin, or EF1a as controls to normalize gene expression analysis. This phase-shifted expression was associated with increased DBP protein levels in *Gα_i3_^−/−^* female *vs*. wild-type livers during the dark phase (ZT12 and ZT18) (Figure 2C). In contrast, the rhythm in hepatic expression of DBP in male mice was comparable in both genotypes at the mRNA (Figure 2B and Supplementary Figure 2B) and protein level (Figure 2D). DBP mRNA levels in male mice showed the same peak as wild-type females peaking at ZT12 similarly as previously described for C57Bl/6 wild-type mice [15]. The sexual dimorphic, phase-shifted expression of DBP became particularly evident when the data from Figure 2A, B were compared between the wild-type (Supplementary Figure 3A) and *Gα_i3_* knockout (Supplementary Figure 3B) of both sexes. In contrast, an analogous re-analysis of the data from Figure 1 revealed rather comparable circadian rhythms in the transcript levels of core molecular clockwork components (with the exception of Per1 and Per2 which displayed slightly to significantly higher transcript levels in females as compared to males of both genotypes) and other clock-regulated genes (like Hlf and Tef) (Supplementary Figure 4). Of note, Gα_i3_ in the liver *per se* showed no rhythmic expression at the mRNA level (data now shown) or the protein level (Supplementary Figure 5). The latter is consistent with a lack of temporal changes of Gα_i_ amounts in the murine SCN [23]. Taken together, hepatic expression of the PAR bZip member DBP in female mice seems to underlie a sexual-dimorphic regulation that is dependent on Gα_i3_.

### Evidence for increased circadian cAMP-CREB signaling in the liver of Gα_i3_ deficient female mice

**Figure 3:**
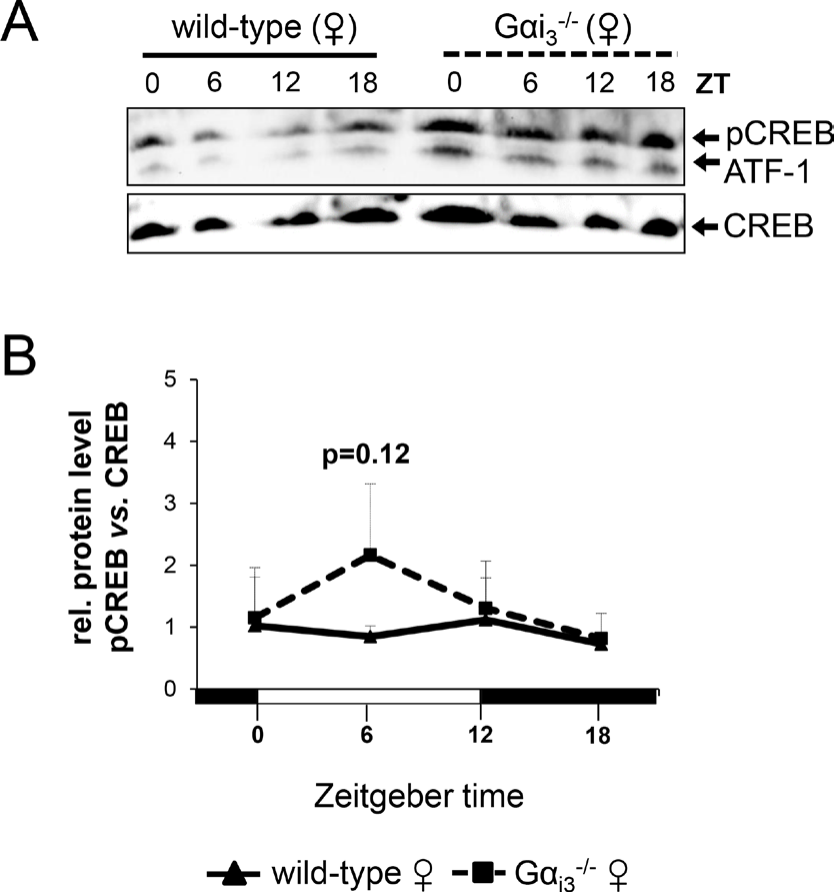
Gα_i3_^-/-^ female mice display increased nuclear pSer133-CREB levels in the liver (**A**) Immunoblot analysis of pSer133-CREB and CREB protein levels in nuclear extracts from livers of female wild-type *vs*. *Gα_i3_*^-/-^ mice. (**B**) Quantitative analysis of relative protein levels (pSer133-CREB/CREB) was performed by ImageJ software. Results are expressed as mean ± s.d. of three independent experiments. Mice were sacrificed and analyzed every six hours at the indicated time points (ZT 0 to ZT 18). ATF1, activating transcription factor 1.

We next addressed the role of Gα_i3_ in the GPCR/Gα_i_ controlled cAMP-CREB signaling pathway as an important regulator of hepatic expression of core clock and clock-regulated genes [16, 17, 19]. Initial analysis of total cell lysates of liver tissue indicated that the cellular levels of pCREB (pSer133-CREB) were higher in *Gα_i3_^−/−^* female mice as compared to corresponding wild-type female controls (Supplementary Figure 6A). In contrast, total pCREB levels were rather comparable between male *Gα_i3_^−/−^* and wild-type mice (as judged by pCREB/CREB signal ratios) thereby following a similar circadian rhythm (Supplementary Figure 6B). Given that pSer133-CREB localizes in the nucleus thereby functioning as transcription factor [31], we next analyzed nuclear extracts isolated from liver tissue. Consistent with the findings above, *Gα_i3_^−/−^* female mice displayed slightly higher nuclear levels of pCREB as compared to wild-type females (Figure 3A, 3B), whereas there was no obvious difference in nuclear pCREB levels between male *Gα_i3_^−/−^* and wild-type mice (Supplementary Figure 7). These findings indicate a sexual-dimorphic inhibitory impact of Gα_i3_ on the cAMP-CREB pathway and suggest that the Gα_i3_-pCREB axis may function as a candidate determinant of female specific expression of DBP. The important role of CREB is emphasized by the observation that selective inhibition of CREB led to a clear decrease in the expression of DBP (Supplementary Figures 9 and 10).

### Elevated transcript levels of the DBP cytochrome P450 target gene Cyp3a11 in Gα_i3_ deficient female mice

**Figure 4:**
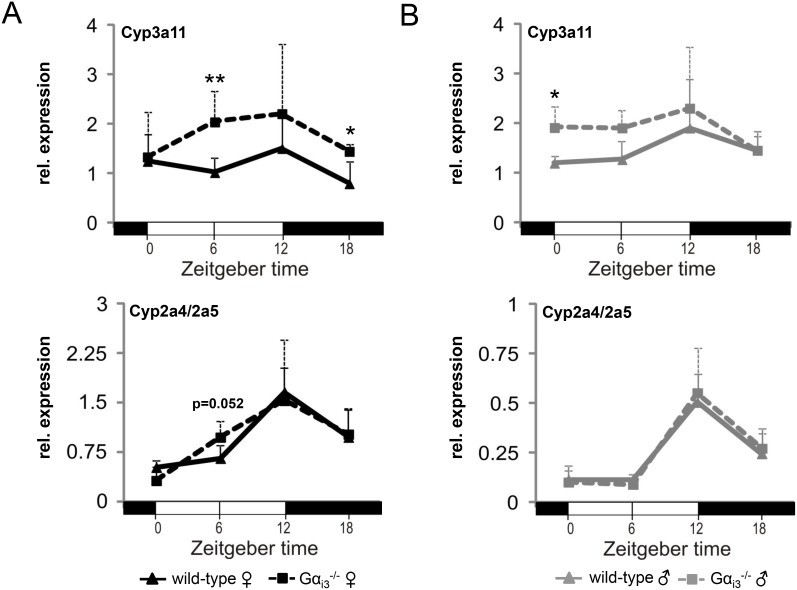
Transcript levels of the DBP target gene and cytochrome P450 family member Cyp3a11 are increased in the liver of *Gα_i3_*^-/-^ female mice Quantitative real-time PCR analysis of rhythmic expression of Cyp3a11 and Cyp2a4/a5 genes in the liver of female (**A**) and male (**B**) Gα_i3_ deficient mice *vs*. wild-type control animals. Mice were sacrificed and analyzed every six hours at the indicated time points (ZT 0 to ZT 18). Transcript levels of Cyp3a11 and Cyp2a4/a5 were normalized to the endogenous control Gapdh. Shown are 2^-ΔCt^ values. Results are expressed as mean ± s.d. of five animals analyzed per genotype and time point (^*^p < 0.05; ^**^p < 0.01 as compared to corresponding wild-type control animals).

DBP drives transcriptionally the rhythmic expression of the xeno- and endobiotic target genes CYP2A4 (Steroid 15α-hydroxylase), CYP2A5 (Coumarin 7-hydroxylase), and CYP3A4 (Cyp3a11 in mouse) [32], which are involved in cholesterol/steroid metabolism or biotransformation of lipophilic xenobiotics. In Gα_i3_ deficient females, transcript levels of Cyp3a11 at ZT06 and ZT18 were significantly higher as compared to levels in the corresponding wild-type females (Figure 4A). In comparison, *Gα_i3_* deficient males displayed levels of Cyp3a11 that were significantly higher at ZT0 as compared to corresponding wild-type males (Figure 4B). In contrast, levels of CYP2A4/CYP2A5 were not different between Gα_i3_ deficient and wild-type mice of both genders (Figure 4A, 4B). Taken together, upon Gα_i3_ deficiency, increased signaling by the cAMP-pCREB axis seems to translate into higher hepatic Cyp3a11 gene expression that was more pronounced in female mice and potentially dependent on transcriptional regulation *via* DBP. The important role of CREB signaling is emphasized by a clear decrease in the expression of DBP and its target gene CYP3A4 in human HepG2 cells upon selective inhibition of CREB (Supplementary Figures 9 and 10).

## DISCUSSION

**Figure 5:**
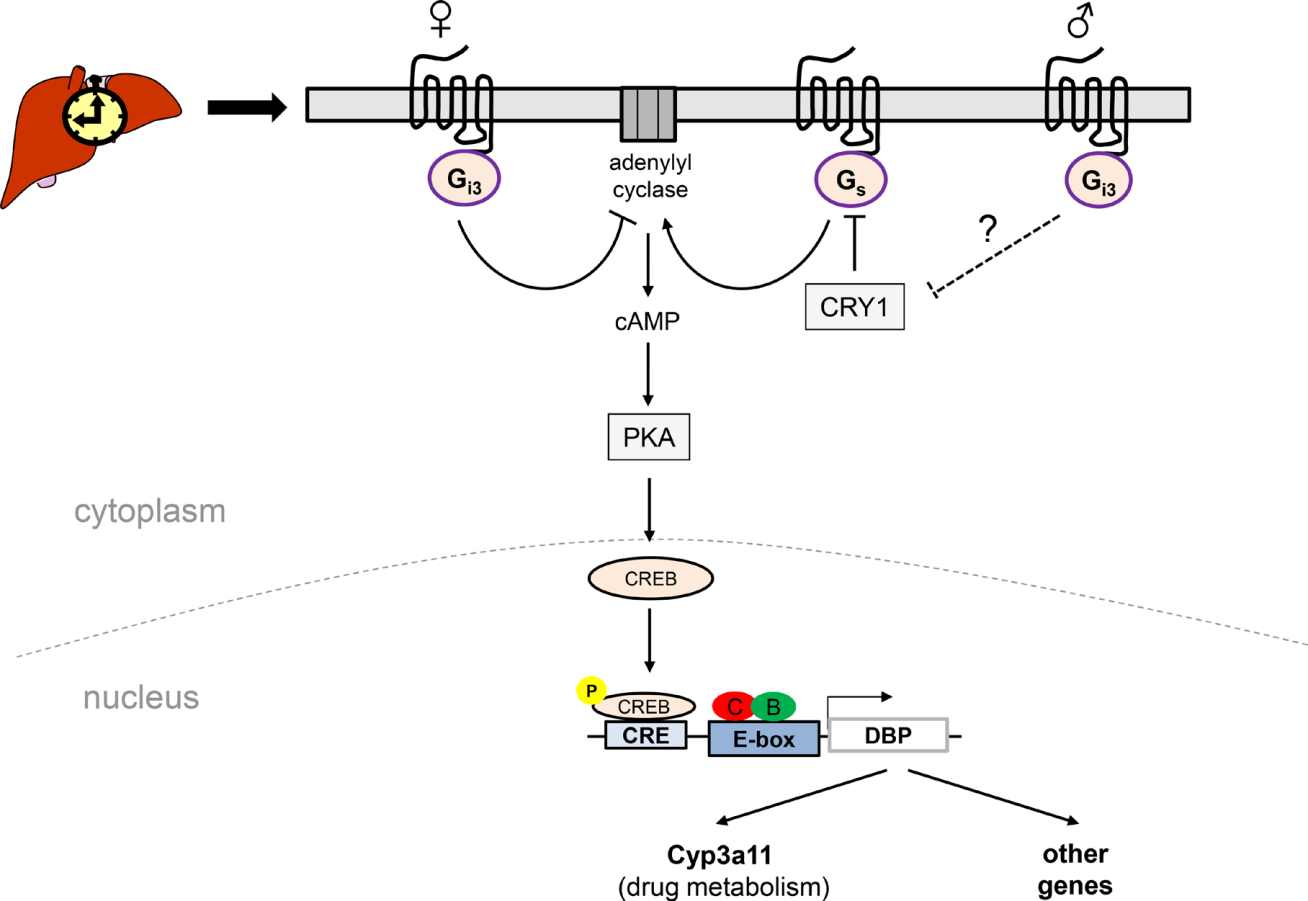
Schematic model suggesting a putative role of the Ga_s_/Gα_i3_-CREB signalling pathways in sexually-dimorphic regulation of DBP expression in female *vs.* male mouse liver In females, loss of Gα_i3_ leads presumably *via* desinhibition of adenylyl cyclase and hence increased pSer133-CREB levels to a phase-advanced and increased expression of DBP in the liver, concomitant with an increase in transcript levels of Cyp3a11, a putative DBP target gene. In *Gα_i3_*^-/-^ males, this signalling pathway may be less active provided that an increased CRY1 expression (as indicated in Figure 1) results in a stronger inhibitory role of CRY1 on cAMP levels (*via* G_s_ inhibition) [24]. B: BMAL1; C: CLOCK; CRE: cAMP responsive element; CREB, cAMP response element binding protein; CRY1, cryptochrome circadian clock 1; Cyp3a11: cytochrome P450, family 3, subfamily a, polypeptide 11; DBP: albumin D-box binding protein; PKA: protein kinase A; G_i3_ and G_s_: heterotrimeric guanine nucleotide binding proteins which upon activation cause a decrease or increase in intracellular cAMP levels, respectively.

This study provides novel insight into the role of the heterotrimeric G protein Gα_i3_ as upstream regulator of the cAMP-pCREB signalling pathway in rhythmic gene expression in the liver. Our data indicate (i) that gene ablation of Gα_i3_ in mice has only modest effects on overall core clock and clock output gene expression and does not affect the period length of clock gene expression; (ii) interestingly, slightly increased levels of nuclear activated CREB (pSer133-CREB) which are linked to a phase-shifted and increased expression of its potential target gene and PAR bZip transcription factor DBP is detectable in livers from Gα_i3_ deficient female mice; (iii) accordingly, the DBP target gene and cytochrome P_450_ family member Cyp3a11 is found at higher transcript levels in the liver of *Gα_i3_^−/−^* female mice. Thus, Gα_i3_ may function as novel regulator of sexual-dimorphic, cAMP-PKA-pCREB driven expression of DBP together with its xenobiotic target genes in the liver (Figure 5). Of note, selective inhibition of CREB led to a clearly decreased expression of DBP and CYP3A4 (human Cyp3a11 homologue) in HepG2 hepatoma cells (Supplementary Figures 9 and 10) further stressing the downstream role of CREB in the regulation of DBP and its target genes.

The phenotypic characterization of Gα_i_ deficient mouse lines has been expanded in the recent years revealing that Gα_i2_ and Gα_i3_ take over gene-specific as well as shared physiological functions in various organs and cell types *in-vivo* [28, 29, 33–41]. The liver expresses both Gα_i2_ and Gα_i3_ with the latter exhibiting an isoform-specific and crucial function in the anti-autophagic action of insulin in hepatocytes [29]. Although a comparative characterization of the hepatic expression of core clock and clock-regulated genes in Gα_i2_ knockout mice is not yet available, we provide evidence that Gα_i3_ deficiency *per se* alters expression of DBP and its target genes in a Gα_i_ isform-specific manner apparently *via* a desinhibited cAMP-pCREB pathway.

Several observations emphasize a critical role of GPCR/G_s_ (cAMP↑) and GPCR/G_i_ (cAMP↓) pathways in the regulation of rhythmic gene expression. Interestingly, Cry1 interacts directly with Ga_s_ at the GPCR level and thereby inhibits adenylyl cyclase activity and accumulation of cAMP and activation of CREB [24]. These findings provided an explanation how the rhythmic expression of Cry1 translates directly into a circadian regulation of cAMP signaling in hepatic glucose metabolism, in particular gluconeogenesis [24]. Moreover, functional inactivation of G_i_-proteins by pertussis toxin [42] or a reduction of cAMP levels by MDL-12330A mediated inhibition of adenylyl cyclase led to a desynchronized circadian gene expression in the SCN and an altered circadian rhythmicity [43, 44]. Here, although the identity of the involved GPCRs in the regulation of circadian signaling is currently only speculative, the G_i_/G_q_-coupled melatonin receptors MT1 and MT2 [45] represent prime candidates given that they play an important role in central clock synchronization. MT1 and MT2 are both expressed in the liver [46] where they display circadian rhythmic changes in gene expression [47]. Interestingly, and consistent with our findings in Figure 2, DBP mRNA levels are phase-advanced in mice lacking either MT1, MT2, or both receptors [46, 48], indicating that these GPCRs are involved in the regulation of DBP expression. However, since the mouse lines analyzed in this study were on the melatonin-deficient C57Bl/6 genetic background [49], backcrossing with mice on the melatonin proficient C3H background is a prerequisite to ultimately determine changes in melatonin receptor signaling and rhythmic hepatic gene expression upon Gα_i3_ deficiency.

In terms of the regulation of DBP expression, previous work showed that maximal and minimal levels of DBP mRNA occur approximately four hours later in the liver as compared to the SCN [50], suggesting that additional regulatory mechanisms (besides transcriptional control *via* E-box elements) must be involved in the control of rhythmic DBP expression in peripheral tissues when compared to the SCN [13, 51–54]. Indeed, treatment of Rat-1 fibroblasts with the adenylyl cyclase activator forskolin was sufficient to increase cAMP-pCREB signalling and trigger circadian expression of DBP [16, 55]. These findings indicate that candidate cAMP inducible promoter elements (CRE) and pCREB binding sites [56], which are regulated by Gα_i3_-mediated signaling, are present in the *Dbp* gene (“direct model”). Indeed, besides the two E-box binding sites (5’-CACGTC-3’), two CRE half sites (5’-CGTCA-3’) can be detected in the promoter region of the murine *Dbp* gene (Singh and Piekorz, unpublished). However, it remains to be determined whether these sites function as pCREB binding elements. Alternatively, one could envision that the subcellular (nuclear *vs.* cytoplasmic) distribution of BMAL-1/CLOCK, that regulates *Dbp* gene expression through E-box binding [57], has become disturbed by Gα_i3_ deficiency in hepatocytes (“indirect model”). So far, however, we failed to detect any obvious differences in nuclear *vs.* cytoplasmic CLOCK protein levels between wild-type and Gα_i3_ deficient hepatocytes at ZT6 and ZT12 (Supplementary Figure 11).

Increasing evidence argues for a role of sexual dimorphism in life span regulation, disease sensitivity, and particularly drug metabolism in females *vs.* males [3, 58–61]. The liver represents a *bona fide* sexual-dimorphic organ with a rhythmic physiology especially in terms of its detoxification function [62, 63]. In these regards, indications for a sexual dimorphic signalling and expression of core clock and clock regulated genes in the liver include based on our study (i) slightly to significantly higher transcript levels of Per1 and Per2 in females *vs*. males (Supplementary Figure 4), (ii) potential differences in pCREB signalling given the levels and rhythms of pSer133-CREB in female *vs.* male animals (Supplementary Figure 6; Figure 3 and Supplementary Figure 7), (iii) a phase-shifted expression of DBP that became particularly evident when comparing wild-type (Supplementary Figure 3A) and *Gα_i3_* knockout (Supplementary Figure 3B) of both sexes, and (iv) a significantly higher circadian expression of CYP2A4/2A5 in females *vs.* males independent of the genotype (Supplementary Figure 8). As summarized in the model in Figure 5, in *Gα_i3_*^−/−^ males, the cAMP-PKA-pCREB signalling pathway may be less active provided that the increased CRY1 expression (Figure 1B) results in a stronger inhibitory role of CRY1 on cAMP levels (*via* G_s_ inhibition) essentially as suggested by Zhang *et al*. [24]. Of note, the increased CRY1 expression in *Gα_i3_*^−/−^ males could be due to a phase shift/earlier onset of Cry1 expression between ZT12 and ZT18, given that ZT intervals of six hours (instead of four hours) have been analyzed in this study.

CYP2A4/2A5 belongs to the cytochrome P_450_ family of monooxygenases, whose expression is under diurnal control by the circadian clock *via* PAR bZip proteins, including DBP. These transcription factors also control the expression of additional enzymes and regulators involved in endobiotic and xenobiotic biotransformation and drug metabolism [15]. In particular, DBP *per se* is able to transcriptionally regulate the circadian accumulation of the target genes CYP2A4 (Steroid 15α-hydroxylase), CYP2A5 (Coumarin 7-hydroxylase), and CYP3A4 (Cyp3a11 in mouse [32]), given that these genes display D-box elements in their promotor regions [64, 65]. CYP2A4/CYP2A5 is involved in cholesterol and bile acid metabolism [64], whereas CYP3A4/Cyp3a11 plays an important role in the metabolism of endogenous steroids and is responsible for biotransformation of approximately 50% of all prescription drugs [65, 66]. The profound role of PAR bZip family members especially in the regulation of drug metabolism and biotransformation [15] becomes particularly obvious in triple knockout mice lacking Dbp, Hlf, and Tef. These animals display a deregulated expression of numerous *cytochrome P_450_* gene family members, an increased sensitivity to xenobiotics, and phenotypic alterations reminiscent of premature aging [12].

In summary, taking our findings in account it remains to be particularly tested whether an increased signaling by the cAMP-pCREB-DBP axis in the liver of Gα_i3_^−/−^ female mice translates into an increased Cyp3a11 activity and therefore improved biotransformation.

## MATERIALS AND METHODS

### Mice

All experiments were performed with Gα_i3_-deficient mice on a C57Bl/6 background with corresponding C57BL/6 wild-type animals used as controls. For breeding and maintenance, mice were kept under specific pathogen-free conditions (SPF) with 12 h light / 12 h dark cycles and free access to food and water at the local animal house of the Heinrich-Heine-University Düsseldorf. The study was performed in accordance with the national and local guidelines on animal care.

### Isolation of liver tissue

Experimental animals (male and female mice between twelve to eighteen weeks of age) were maintained under standard conditions with 12 h light / 12 h dark cycles. Animals were killed by cervical dislocation and livers were obtained at the Zeitgeber times (ZT) 0, 6, 12, and 18 (ZT 0: Light ON; ZT 12: Light OFF) and immediately frozen in aliquots in liquid nitrogen for further analysis.

### RNA isolation and quantitative real-time PCR (qRT-PCR)

Total RNA from liver tissue was extracted using RNeasy mini kit spin columns (QIAGEN). RNA purity and concentration was determined using a NanoDrop spectrophotometer. One μg of liver RNA was reverse-transcribed using oligo(dT)_15_ primers and the ImProm™ II Reverse Transcription System (Promega) according to the manufacturer's specifications. Relative quantification of mRNA was carried out using quantitative real-time PCR (qRT-PCR; 7500 Real-Time PCR System; Applied Biosystems) and specific TaqMan probes (Applied Biosystems) for core clock genes and clock regulated genes [67]. Expression of target sequences was normalized to an endogenous control, glyceraldehyde-3-phosphate dehydrogenase (GAPDH: Part No.: 4331182, Assay-ID: Mm99999915_g1). For further normalization to other endogenous controls, β-Actin and EF1a were used (Schneider *et al*., 2014). qRT-PCR for cytochrome P_450_ isoforms was performed using TaqMan probes for Cyp3a11 (Part No.: 4331182, Assay-ID: Mm00731567_m1) and Cyp2a4/Cyp2a5 (Part No.: 4331182, Assay-ID: Mm00487248_g1).

### Total cell lysates and immunoblot analysis

Total cell lysates from mouse liver were prepared as described [68] with minor modifications. Briefly, snap-frozen liver tissue was homogenized with an Ultra-Turrax for 30–60 s at 4° C in 800 μl of lysis buffer (50 mM Tris-HCl, pH 8.0; 150 mM NaCl; 1 mM EDTA; 1% Triton-X100; 0.5% deoxycholic acid; 1% SDS) supplemented with cocktails of protease inhibitors (Roche) and phosphatase inhibitors (Sigma-Aldrich)]. After sonication for 45 seconds, tissue homogenates were cleared by a centrifugation step (30.000 × g, 15 min). Lysates were subjected to protein electrophoresis employing 8.5–10% SDS-acrylamide gels or 6 M urea/SDS-PAGE gels and blotted onto nitrocellulose membrane [29]. Membranes were blocked in 5% non-fat dry milk and incubated with primary antibodies at 4° C overnight (anti-Gα_i3_, Santa Cruz sc-262, 1:1000; anti-DBP, abcam ab22824, 1:1000; anti-phospho-CREB [pSer133-CREB], Cell Signaling 87G3 #9198, 1:1000; anti-CREB, Cell Signaling 48H2 #9197, 1:1000; anti-GAPDH, abcam ab8245, and anti-Actin C4, Millipore, both 1:5000). The anti-phospho-CREB antibody recognizes the phosphorylated form of CREB (pSer133-CREB) and the activating transcription factor-1 (ATF-1). Nuclear and cytoplasmatic CLOCK levels were detected using a primary antibody from Cell Signaling (D45B10, rabbit mAb #5157; 1:1000). Antibodies against total Histone (Cell Signaling #9715, 1:1000) or Actin (C4, Millipore, 1:5000) was employed to control protein loading of nuclear and cytoplasmatic fractions, respectively. Following three washing steps membranes were incubated with a horseradish peroxidase (HRP)-conjugated anti-rabbit IgG antibody (Cell Signaling #7074) or a HRP-conjugated polyclonal rabbit-anti-mouse IgG (Dako P0161) both at a dilution of 1:5000 for 60 min at room temperature. Protein signals were visualized by the ECL detection system (GE Healthcare) and images were collected using an INTAS chemostar imager.

### Isolation of nuclear proteins from mouse liver

Nuclear proteins from 12–18 weeks old Gα_i3_^−/−^ and wild-type livers (ZT 0, 6, 12, and 18) were prepared essentially as described [69]. In brief, 0.5–0.6 g of liver tissue was washed in phosphate buffered saline (PBS) buffer (pH 7.4) and disintegrated in 4 ml of ice-cold buffer A (250 mM sucrose, 5 mM MgCl_2_, 10 mM Tris-HCl [pH 7.4]) using 5 ml syringe plungers and 40 μm pore size cell strainers. Samples were centrifuged at 800 × g for 10 min at 4° C. The supernatant was collected as cytoplasmatic fraction and stored at −80° C for further analysis. The pellet was gently resuspended in 14 ml of ice-cold buffer A and centrifuged at 1000 × g for 10 min. The resulting pellet was resuspended in 1 ml of ice-cold buffer B (2.0 M sucrose, 1 mM MgCl_2_, 10 mM Tris-HCl [pH 7.4]) followed by a centrifugation step at 16.000 × g and 4° C for 30 min. The final pellet was resuspended in Laemmli SDS sample buffer and kept at −80° C for further analysis. Alternatively, subcellular fractionation of liver tissue was performed using the Nuclear Extract Kit (Version D3) from Active Motif, Inc. (La Hulpe, Belgium) essentially as decribed by the manufacturer.

### Densitometric analysis

Quantitative immunoblot analysis was performed using ImageJ software (http://imagej.nih.gov/ij/).

### Statistical analysis

Results are given as mean ± s.d. To evaluate statistical significance, Student's *t*-tests or ANOVA tests were performed. *P*-values ≤ 0.05 were considered statistically significant.

## SUPPLEMENTARY MATERIALS FIGURES



## Abbreviations

ATF1: activating transcription factor 1
Cyp3a11: cytochrome P_450_: family 3: subfamily a: polypeptide 11
CREB: cAMP responsive element binding protein
DBP: albumin D-box binding protein
Gα_i_: inhibitory G protein alpha subunit
G_i_: heterotrimeric Gα_i_βγ protein
PAR bZip: PAR-domain basic leucine zipper
PKA: protein kinase A
qRT-PCR: quantitative real-time PCR
ZT: Zeitgeber time.
