# Supplementary material for: Gαi3 signaling is associated with sexual dimorphic expression of the clock-controlled output gene Dbp in murine liver
Source: Oncotarget. 2018 Jul 13;9(54):30213–24. doi: 10.18632/oncotarget.25727 (PMC6084400; doi:10.18632/oncotarget.25727)
Supplement: Supplementary file 1 [file oncotarget-09-30213-s001.pdf]

$G\alpha_{i3}$ signaling is associated with sexual dimorphic expression of the Clock-controlled output gene *Dbp* in murine liver

SUPPLEMENTARY MATERIALS

SUPPLEMENTARY EXPERIMENTAL PROCEDURES

Isolation of tail fibroblasts

The isolation and culture of murine tail fibroblasts was performed as described [2]. Tails (1–2 cm lengths) were washed in 70% ethanol and incubated on ice for 1 h in complete Dulbecco's modified Eagle medium supplemented with 10% fetal bovine serum and penicillin-streptomycin. Following alternate washes in ice cold PBS containing penicillin-streptomycin and 70% ethanol, tails were minced into small pieces and digested overnight with collagenase type II (400 units/ml; 1600 units per tail) in a humidified incubator (7% CO_2). Cells were isolated by repeated pipetting, filtered through a 70 µm nylon cell strainer, centrifuged at 1000 rpm for 5 min, and finally resuspended in complete medium. Cells were grown on 60 mm dishes to a confluency of 80–90% prior to passaging on 20 cm culture plates (1×10^6 cells/plate). Tail fibroblasts between passages 1 and 5 were analyzed.

Production of lentiviral vectors

Lentiviral vectors were produced by polyethyleneimine (PEI, Sigma-Aldrich, Germany) based transfection of HEK293T cells with 6 µg of an HIV1 packaging plasmid (pCMVΔR8.2), 6 µg of the envelope vector (pMD2.G), and 6 µg of the vector plasmid with the Bmal1-Luciferase reporter gene [1, 3]. Viral supernatants were harvested 48 hours later, passed through a 0.45-µm filter (Sartorius AG, Germany), and then used to transduce primary tail fibroblasts. After 24 h the viral supernatant was replaced with fresh medium.

Determination of period length in primary tail fibroblasts by Bmal-luciferase real-time bioluminescence monitoring

Lentivirally transduced primary tail fibroblasts stably expressing a Bmal1-Luciferase reporter gene were grown in Dulbecco's modified Eagle's medium (DMEM) supplemented with 10% fetal calf serum (FCS) and 1% penicillin/streptomycin (Life Technologies). Confluent cells were trypsinized and 5×10^5 cells were seeded into 35 mm culture dishes one day before real-time bioluminescence monitoring was initiated. Cellular clocks were synchronized with 100 nM dexamethasone for 30 min. After synchronization the medium was replaced with phenol red-free DMEM (Life Technologies) supplemented with 10% FCS and 100 µM luciferin. Real-time bioluminescence was monitored in a light-tight incubator using photomultiplier tube detector assemblies (LumiCycle, Actimetrics). Phase, amplitude, and period length of circadian oscillations were determined with the LumiCycle Analysis software.

Quantitative real-time PCR analysis of DBP and CYP3A4 expression in human HepG2 hepatoma cells

HepG2 cells were grown in Dulbecco's modified Eagle's medium (DMEM) supplemented with 10% fetal calf serum (FCS) and 1% penicillin/streptomycin (Life Technologies). The adenylyl cyclase activator Forskolin and the selective and *in-vivo* well tolerated CREB inhibitor 666-15 [4] were obtained from Sigma-Aldrich and Tocris, respectively. Total RNA was extracted using the Universal mRNA/miRNA Purtification Kit (roboklon). One µg of liver RNA was reverse-transcribed using oligo$(dT)_{15}$ primers and the ImProm™ II Reverse Transcription System (Promega) according to the manufacturer's specifications. Relative quantification of mRNA was carried out using SYBR® Green-based real-time RT-PCR (7500 Real-Time PCR System; Applied Biosystems) using the QuantiTect® primer assay for DBP (Hs_DBP_1_SG) and CYP3A4 (Hs_CYP3A4_1_SG) from Qiagen and the Fast SB qPCR Master Mix plus ROX (roboklon). For normalization β-Actin mRNA levels were determined.

REFERENCES

1. Nagoshi E, Saini C, Bauer C, Laroche T, Naef F, Schibler U. Circadian gene expression in individual fibroblasts: cell-autonomous and self-sustained oscillators pass time to daughter cells. Cell. 2004; 119:693–705.
2. Salmon AB, Murakami S, Bartke A, Kopchick J, Yasumura K, Miller RA. Fibroblast cell lines from young adult mice of long-lived mutant strains are resistant to multiple forms of stress. Am J Physiol Endocrinol Metab. 2005; 289:E23–9.
3. Schneider R, Linka RM, Reinke H. HSP90 affects the stability of BMAL1 and circadian gene expression. Journal of Biological Rhythms. 2014; 29:87–96.
4. Li BX, Gardner R, Xue C, Qian DZ, Xie F, Thomas G, Kazmierczak SC, Habecker BA, Xiao X. Systemic inhibition of CREB is well-tolerated *in vivo*. Sci Rep. 2016; 6: 34513. https://doi.org/10.1038/srep34513.

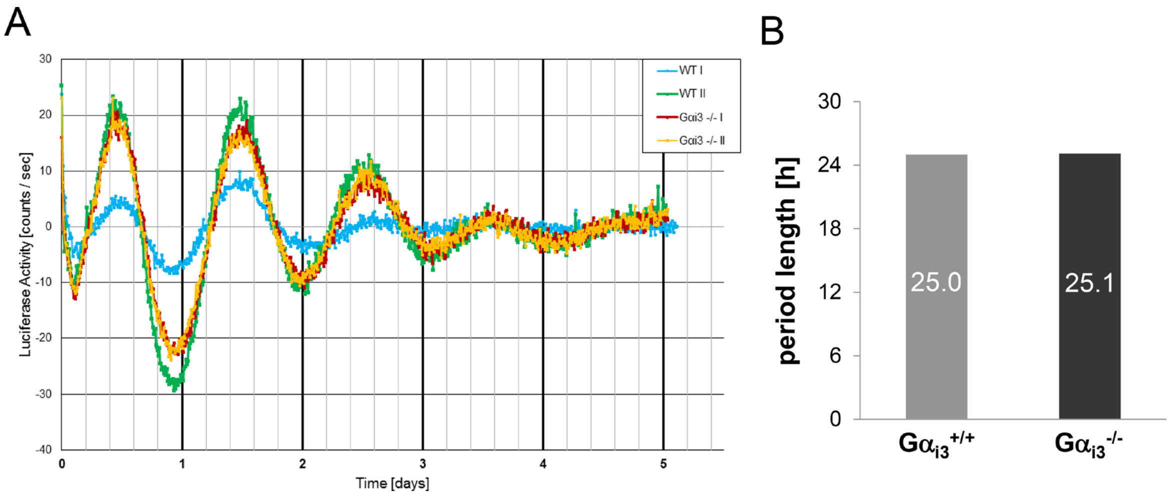

Supplementary Figure 1: The period length of primary tail fibroblasts isolated from Gα_{i3} deficient female mice is comparable to the period length of wild-type control fibroblasts. Lentivirally transduced primary tail fibroblasts stably expressing a Bmal1-Luciferase reporter gene were subjected to real-time bioluminescence monitoring (**A**) as described in the supplementary experimental procedures. Fibroblasts were transduced and analyzed giving rise to a period length that was comparable between wild-type and $G\alpha_{i3}^{-/-}$ fibroblasts (mean of two independent lines analyzed per genotype) (**B**).

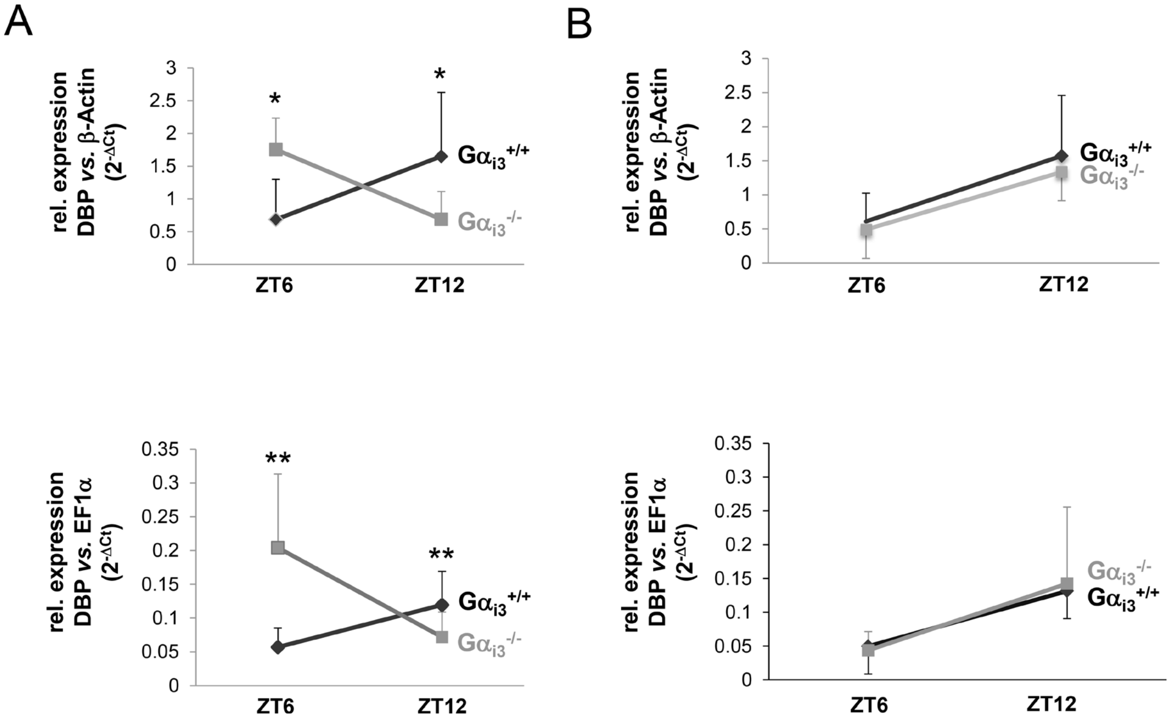

Supplementary Figure 2: Expression analysis of DBP transcript levels in the liver of female $G\alpha_{i3}^{-/-}$ mice using different normalization controls. (**A, B**) Quantitative real-time PCR analysis of rhythmic expression of DBP mRNA in the liver of female (A) and male (B) $G\alpha_{i3}$-deficient mice as compared to wild-type control animals. β-Actin (upper panels) and EF1a (lower panels) were used as normalization controls. Mice were sacrificed and analyzed at ZT6 and ZT 12. Results are expressed as mean ± s.d. of six animals analyzed per genotype and time point ($^{*}p < 0.05$, $^{**}p < 0.01$ as compared to wild-type control animals).

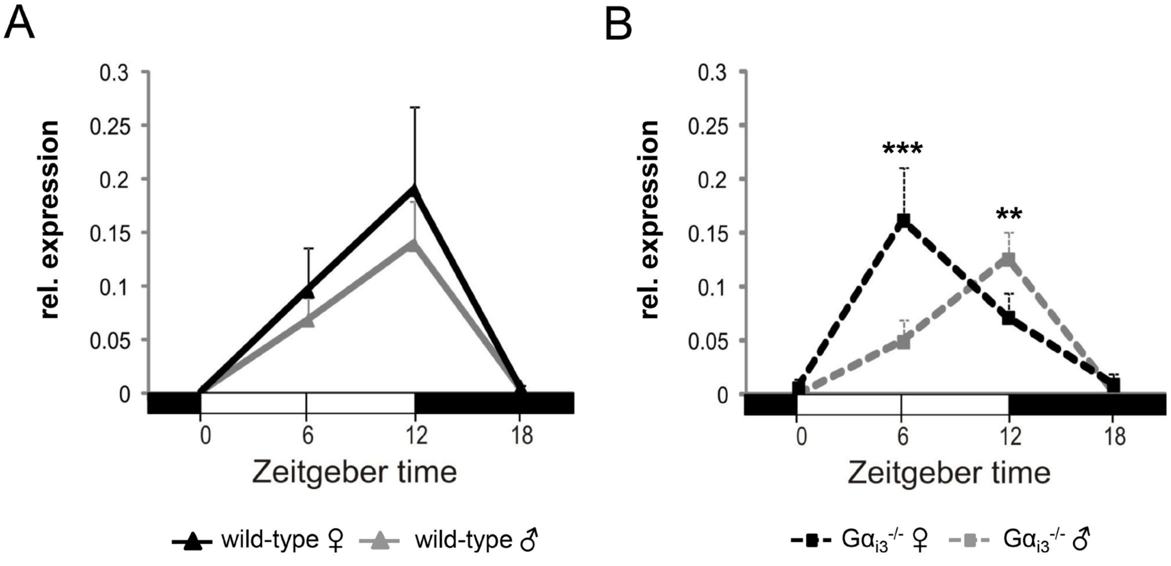

Supplementary Figure 3: Loss of $G\alpha_{i3}$ reveals a sexual-dimorphic expression of the albumin D-box binding protein (DBP) in murine liver. Quantitative real-time PCR analysis of circadian expression of DBP mRNA in the liver of female *vs.* male wild-type animals (**A**) and female *vs.* male $G\alpha_{i3}$ deficient animals (**B**). Mice were sacrificed and analyzed every six hours at the indicated time points (ZT 0 to ZT 18). Dbp transcript levels were normalized to the endogenous control Gapdh. Shown are $2^{-\Delta Ct}$ values. Data are expressed as mean ± s.d. of six animals analyzed per genotype and time-point ($^{**}p < 0.01$, $^{***}p < 0.001$).

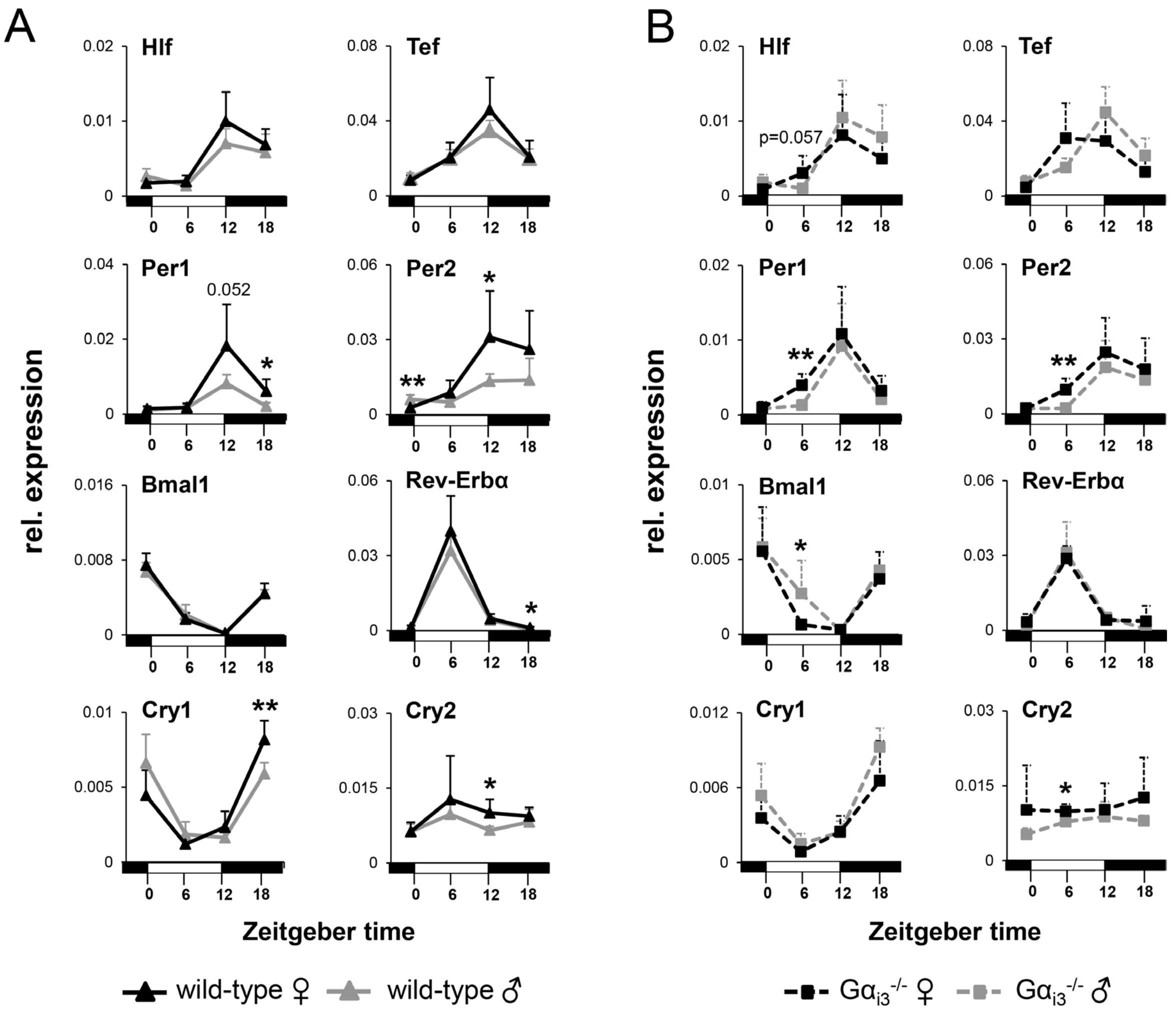

Supplementary Figure 4: Analysis of rhythmic and sex-specific expression of core clock genes and clock-regulated genes in the liver of wild-type and *$G\alpha_{i3}^{-/-}$* mice. Quantitative real-time PCR analysis of circadian expression of DBP mRNA in the liver of female *vs.* male wild-type mice (**A**) and female *vs.* male $G\alpha_{i3}$ deficient animals (**B**). Mice were sacrificed and analyzed every six hours at the indicated time points (ZT 0 to ZT 18). Transcript levels of the indicated genes were normalized to the endogenous control Gapdh. Shown are $2^{-\Delta Ct}$ values. Results are expressed as mean ± s.d. of six animals analyzed per genotype and time point ($^{*}p < 0.05$, $^{**}p < 0.01$ as compared to wild-type control animals).

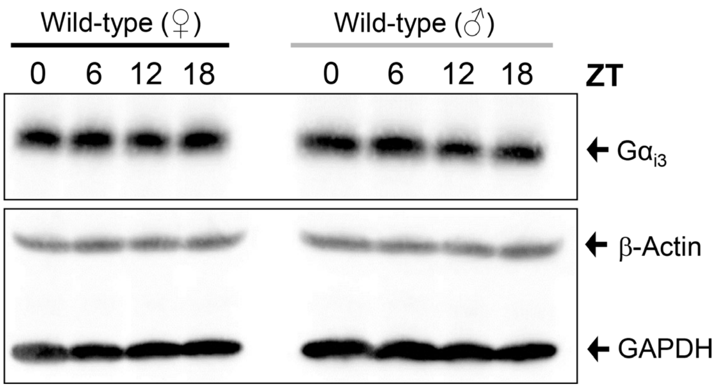

Supplementary Figure 5: Comparable rhythmic Gα$_{i3}$ protein levels in the liver of male and female wild-type mice. Immunoblot analysis of total protein lysates from the liver of wild-type male and female mice employing a Gα$_{i3}$ isoform-specific antibody. GAPDH and β-Actin were employed as normalization controls. Mice were sacrificed and analyzed every six hours at the indicated time points (ZT 0 to ZT 18).

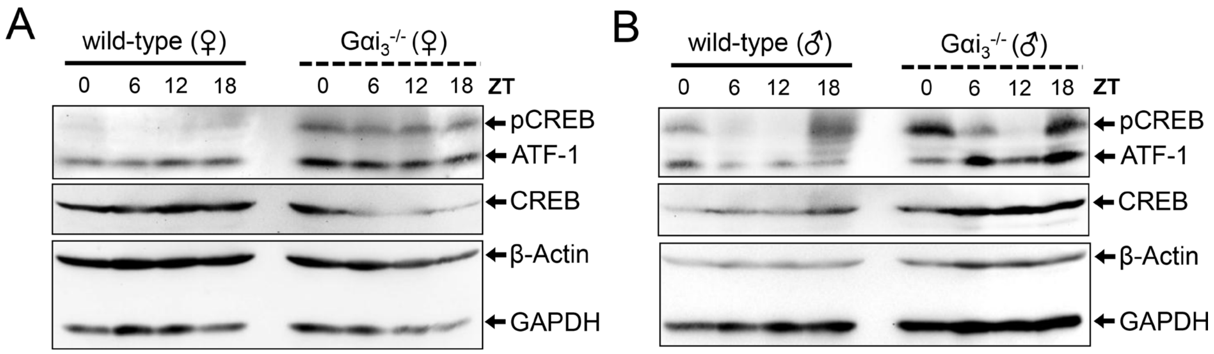

Supplementary Figure 6: *Gα$_{i3}^{-/-}$* female mice display increased cellular pSer133-CREB levels in the liver. Immunoblot analysis of pSer133-CREB levels and CREB expression in total cell lysates from livers of female (**A**) and male (**B**) wild-type *vs*. *Gα$_{i3}^{-/-}$* mice. GAPDH and β-Actin were detected as normalization controls. Mice were sacrificed and analyzed every six hours at the indicated time points (ZT 0 to ZT 18). ATF1, activating transcription factor 1.

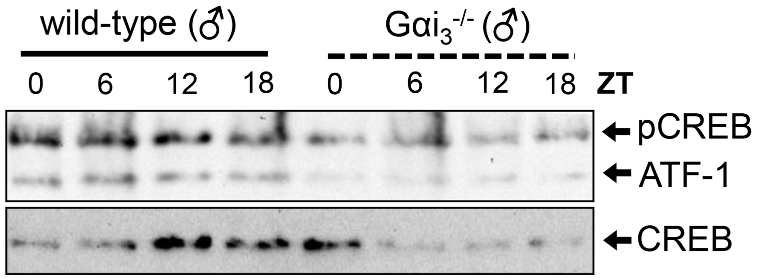

Supplementary Figure 7: Comparable nuclear pSer133-CREB / CREB levels in the liver of $G\alpha_{i3}^{-/-}$ and wild-type male mice. Immunoblot analysis of pSer133-CREB and CREB protein levels in nuclear *vs.* cytoplasmatic extracts from livers of male wild-type *vs.* $G\alpha_{i3}^{-/-}$ mice. Animals were sacrificed and analyzed every six hours at the indicated time points (ZT 0 to ZT 18). ATF1, activating transcription factor 1.

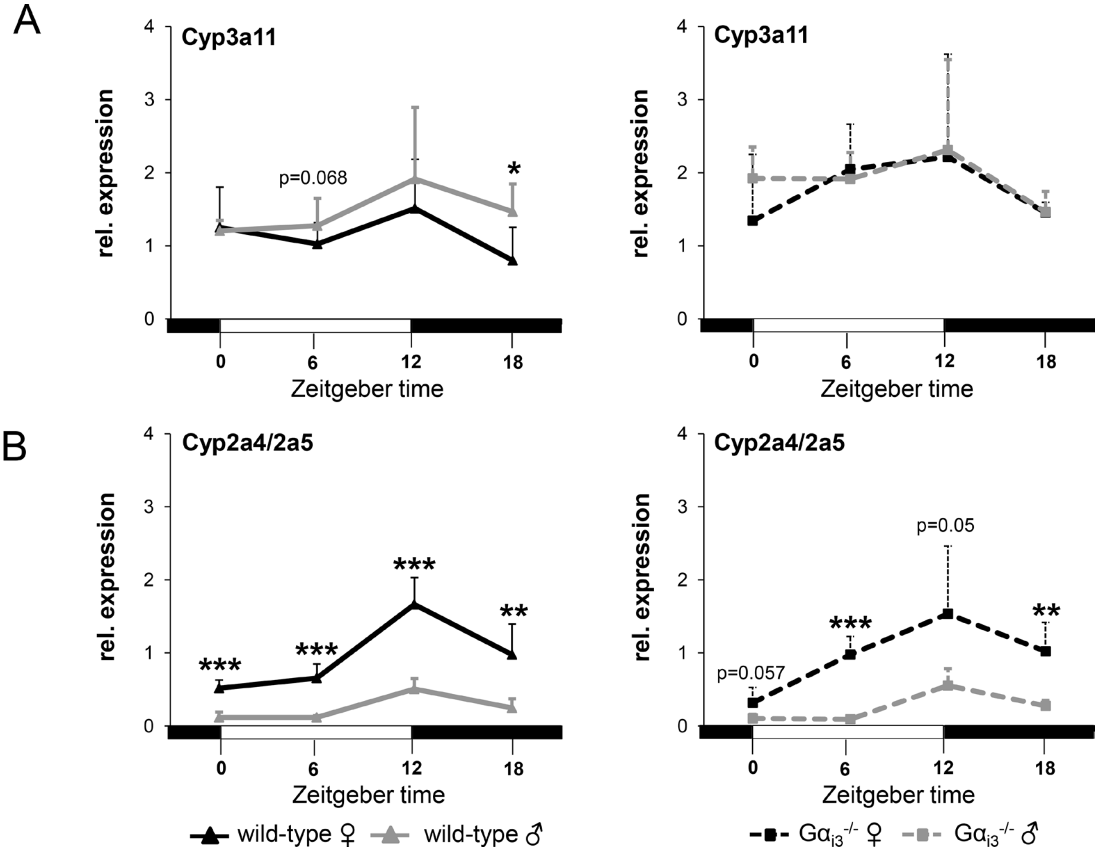

Supplementary Figure 8: Rhythmic and sex-specific expression of Cyp3a11 and Cyp2a4/a5 genes in the liver of wild-type and $G\alpha_{i3}^{-/-}$ mice. Quantitative real-time PCR analysis of circadian expression of Cyp3a11 (**A**) and Cyp2a4/a5 (**B**) mRNAs in the liver of female *vs.* male wild-type animals (left panels) and female *vs.* male $G\alpha_{i3}$-deficient animals (right panels). Mice were sacrificed and analyzed every six hours at the indicated time points (ZT 0 to ZT 18). Cyp3a11 and Cyp2a4/a5 transcript levels were normalized to the endogenous control Gapdh. Shown are $2^{-\Delta Ct}$ values. Results are expressed as mean ± s.d. of five animals analyzed per genotype and time point ($^{*}p < 0.05$, $^{**}p < 0.01$, $^{***}p < 0.001$ as compared to wild-type control animals).

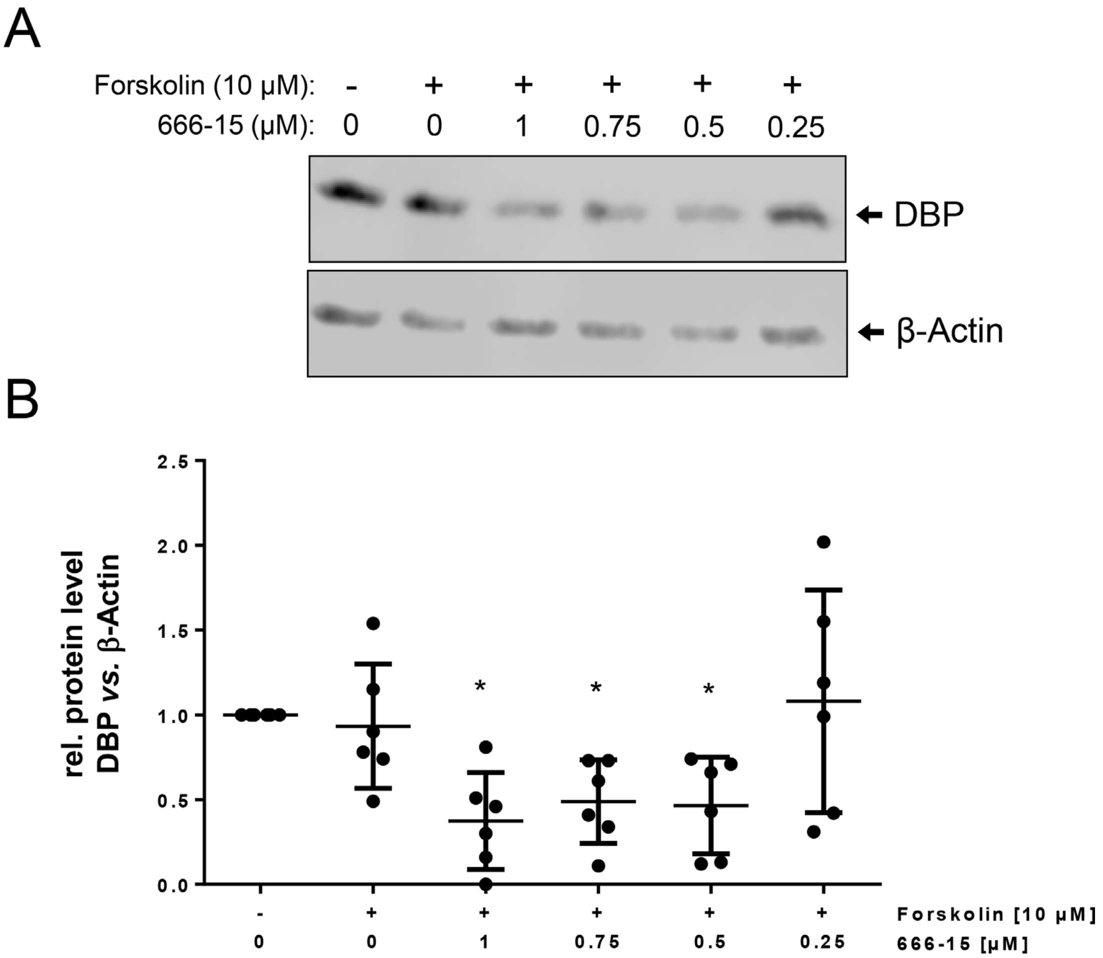

Supplementary Figure 9: Decreased DBP protein levels upon inhibition of CREB in human HepG2 liver cells. (**A**) Immunoblot analysis and (**B**) subsequent ImageJ based quantification of DBP protein levels in HepG2 cells upon 666-15 mediated inhibition of CREB. Cells were pre-treated with the selective CREB inhibitor 666-15 [4] (0.25–1.0 μM) for 1h followed by treatment with 10 μM Forskolin for 5 h. DBP protein levels were normalized to the endogenous control β-Actin. Results in (B) are expressed as mean ± s.d. of six experiments ($^{*}p < 0.05$ as compared to untreated cells).

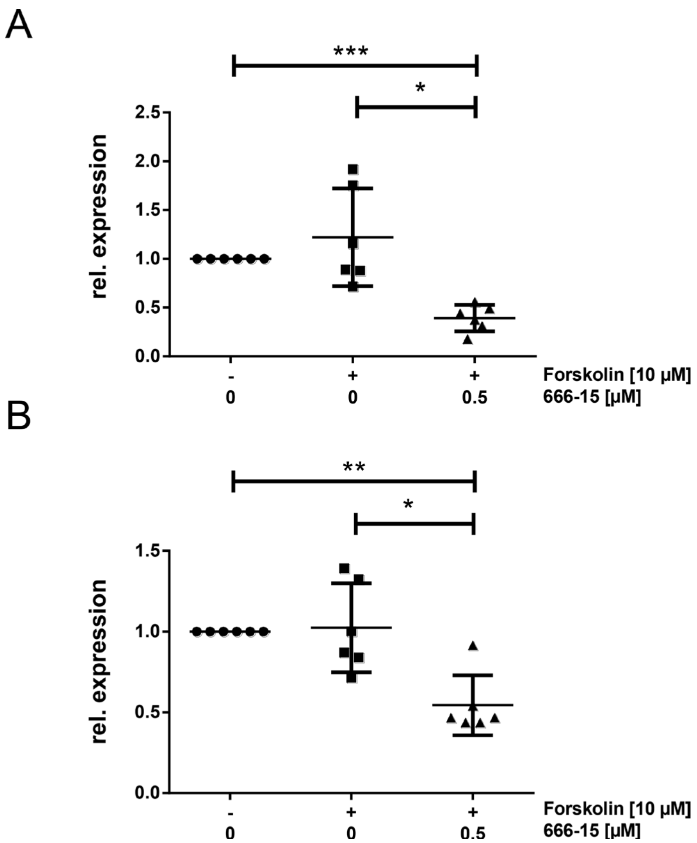

Supplementary Figure 10: Decreased DBP and CYP3A4 mRNA levels upon inhibition of CREB in human HepG2 liver cells. Quantitative real-time PCR analysis of DBP (**A**) and CYP3A4 mRNA levels (**B**) in HepG2 cells upon inhibition of CREB. Cells were pre-treated with the selective CREB inhibitor 666-15 [4] (0.5 µM) for 1h followed by treatment with 10 µM Forskolin for 5 h. Dbp and CYP3A4 transcript levels were normalized to the endogenous control β-Actin. Shown are $2^{-\Delta Ct}$ values. Results are expressed as mean ± s.d. of six experiments ($^{*}p < 0.05$, $^{**}p < 0.01$, $^{***}p < 0.001$ as compared to untreated cells).

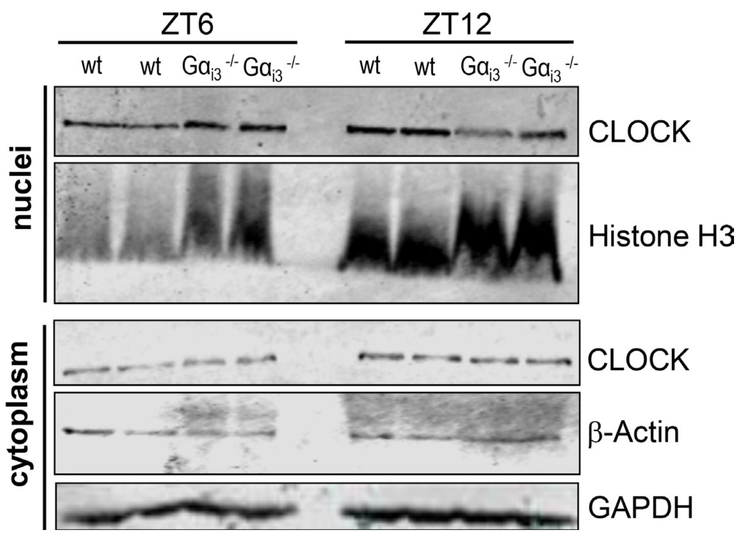

Supplementary Figure 11: Levels of nuclear CLOCK are not affected in the liver of G$\alpha_{i3}^{-/-}$ female mice. Immunoblot analyses of nuclear proteins from the liver of female Gα_{i3} deficient mice *vs*. wild-type control animals. An antibody recognizing the core clock regulator CLOCK was employed. Total Histone and β-Actin/GAPDH levels were detected to normalize protein loading of nuclear and cytoplasmatic fractions, respectively. Mice were analyzed at ZT 6 and ZT 12.
